# Editorial Comment: Effects of spongioplasty on neourethral function following hypospadias repair: an experimental study on rabbits

**DOI:** 10.1590/S1677-5538.IBJU.2019.0453.1

**Published:** 2020-02-20

**Authors:** Antonio Macedo

**Affiliations:** 1 CACAU-NUPEP Departamento de Urologia São PauloSP Brasil Departamento de Urologia, CACAU-NUPEP, São Paulo, SP, Brasil; 2 Universidade Federal de São Paulo Departamento de Pediatria São PauloSP Brasil Departamento de Pediatria, Universidade Federal de São Paulo, São Paulo, SP, Brasil

## COMMENT

Authors have evaluated on an experimental study on rabbits the role of spongioplasty after urethral plate tubularization (Duplay technique) by assessing intraluminal urethral plate pressure and flow rate (ex vivo) (1). Two similar groups were created with exception for spongioplasty added to Duplay urethroplasty. Both groups presented similar results with no statistical differences. The fistula rate was higher in the no spongioplasty group (14.29%).

Barrier layers prevent fistula occurrence as shown here, but this is a well known concept that does not require further experimentation. The authors hypothesized that spongioplasty would compress or impact on urethra distensibility or compliance, which is not related to spongioplasty itself but to the degree of tension when suturing both wings of spongious tissue. This is a singularity of the procedure that can not be randomized and compared, because it is a surgical maneuver based on personal experience.

The authors are to be congratulated for the methodology of their work but in a translational world the surgeon awaits experimental studies to advance on relevant questions after clinical procedure. In hypospadias repair, the interposition of well vascularized tissue is desired and one can not omit using viable spongious tissue that has the advantage of being situated adjacent to the neourethra.
